# Evaluating Spatial Identity Based on Climate Adaptation in Small Cities

**DOI:** 10.3390/ijerph20010713

**Published:** 2022-12-30

**Authors:** Tao Luo, Zijing Zhang, Xinchen Hong, Yanyun Wang, Xuewei Zhang

**Affiliations:** 1 School of Architecture and Urban-Rural Planning, Fuzhou University, Fuzhou 350108, China; 2 Fujian Geological Remote Sensing and Geographic Information Service Center, Fuzhou 350011, China

**Keywords:** urban form, spatial identity, climate adaptation, coupling coordination, small city

## Abstract

Urban spatial identity is declining in Chinese cities overall due to urbanization, which is attracting increasing attention from the government. Research gaps include systematically comparing urban identities based on causes and manifestations in small cities. We developed a framework for estimating spatial identity from the perspective of climate adaptation, which is based on the relationship between regional climate and spatial form. Five small cities were selected in China: Wu’an, Qingcheng, Jintang, Changxing, and Lianjiang. Our findings suggest that (1) typical indicators include impervious surface rate, green coverage rate, water surface rate, average story number, and total gross floor area, contributing to morphological characteristics influenced by climate drivers; (2) for the hot humid climate zones, the city with the highest level of spatial identity is in Jintang, followed by Lianjiang and Changxing; and for the cold climate zones, the level of spatial identity in Qingcheng was higher than in Wu’an. This can contribute to the understanding and methodology of spatial identity based on climate adaptation in small cities.

## 1. Introduction

UNDESA’s report indicated that 55% of the world’s population lives in urban environments in 2018 [1]. Urban spaces have become the primary place for humans to live. Related research has concluded that most urban growth will occur in small cities [2], so the importance of small cities is becoming more and more evident. Spatial identity is increasingly recognized as one of the essential assets of cities [3]. Unfortunately, technological advancements in building processes brought about changes in urban space [4], which further contribute to the decrease of urban identity [5]. This phenomenon is especially evident in China because of its rapid urbanization. Accordingly, the current policy in China emphasizes the importance of strengthening urban identity for the urban environment’s construction. Compared to China’s large and medium-sized cities, small cities retain more characteristic spaces; thus, their spatial identities are more valuable to study.

Research on urban morphology and other related topics has paid attention to urban spatial identity. Research related to urban identity began to develop after the 1950s, as the built environment of the emerging city was influenced by modernist planning strategies and appeared more homogeneous and proximate [6]. Related research contributes three approaches to describing spatial identity: experience, discourse, and location [7]. Landscape, culture, and economy are used to explore the components of urban spatial identity [8].

The understanding of spatial identity remains susceptible to ambiguity due to its multiple variants and different usages. Numerous variations of the term spatial identity are used in different ways. From a perceptual perspective, it can be considered a sense of space, a spatial image, a perception of place, etc. [3]. However, perception is not the focus of this study. Previous research argues that spatial identity is often considered the “objective reality” and is thus, opposed to the image (how a place is perceived externally) [9]. In our study, identity is “the distinguishing character or condition of a person or a thing”, according to Webster’s Ninth New Collegiate Dictionary (1983) and is also often associated with individuality and commonality [10]. We also consider “identity” as a matter of differences, as “the things that give a city its identity are the things that make it different from other cities” [11]. In conclusion, the term “urban spatial identity” can be recognized to describe a city’s distinctive, physical, environment features [12]. These features are clearly distinguished from those of other cities due to factors such as nature and culture. This study focused on urban spatial identity under climatic factors.

Urban space is considered a dynamic and adaptive reaction to the expectations of residents, topography, climate, and culture [13]. Among them, climate is an essential factor relating to spatial form. It is a decisive factor in the creation of settlements and a powerful leading force in implementing spatial measures at the level of government [14,15]. On the one hand, climate affects residents’ behavior, the cultural background of the group, and environmental awareness, contributing to alterations in the structure’s design [16,17]. There is a long tradition of considering climate issues in urban design, dating back more than 2000 years [18]. Currently, there are also many places where urban design has considered solar rights and wind protection [19]. On the other hand, urban space also influences local climate in functions and shapes. Four drivers affect urban climate, including urban structure, land cover, urban fabric, and urban metabolism [20]. For example, vegetation changes in land cover can potentially influence temperature [21]. Furthermore, residents play a role in the bidirectional adjustment of climate and space as a medium. This is reflected in the climate adaptation programs of historical buildings and climate-adaptive solutions in urban forms [22].

**Figure 1:**
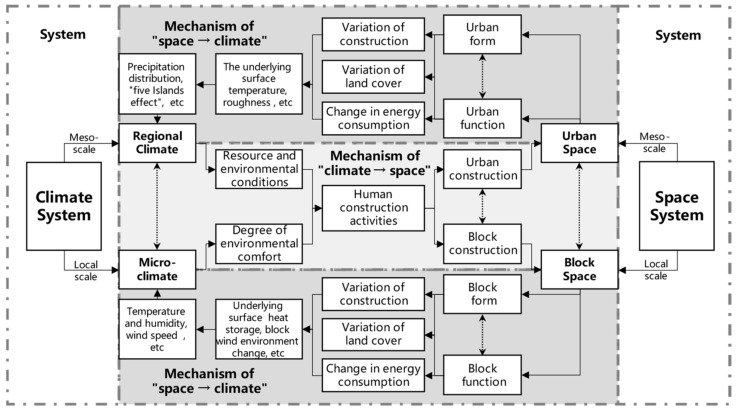
Coupled modelling regional climate and urban spatial systems.

Therefore, we summarize the relationship between the roles of climate and space (see Figure 1). There is an interaction between climate and urban space on the mesoscale (for a municipal region or city planning) and the local scale (for a neighborhood or block planning) [23,24]. The spatial and climatic systems are in constant conflict and coordination, gradually converging to a stable condition. Therefore, we believe that the level of urban spatial identity can be assessed according to the extent of this coupled coordination.

Generally speaking, field surveys and literature reviews are the standard evaluation methods used to assess urban spatial identity [8]. Many studies focus on evaluating urban spatial identity based on public perception [8,10]. However, too much attention is paid to the superficial perspective, and little is paid to investigating the intrinsic causes and mechanisms [11]. Interviews and observations are considered viable methods for research in limited areas because of time and cost constraints [7,25,26]. In order to improve the scientific nature of the evaluation results, some scholars have introduced mathematical modelling methods [8]. The collection and analysis of a large amount of spatial data through RS, GIS, and GPS technologies also facilitates the relevant investigation of urban spatial identity and systematic comparison between several areas.

In addition, small cities have for too long been ignored by urban theorists, and this is no exception in the study of spatial identity [27]. The spatial identity of the central cities has received more attention from researchers [28,29,30]. Furthermore, related research on urban spatial identity often focuses on unique places such as public urban spaces [28,29,31], the coastal region [10], and urban heritage [32]. However, cities are not homogenized entities [27]. A study focused specifically on small cities would be more applicable to them.

Thus, this study aimed to compare and analyze the spatial identity of small cities regarding the effects of climate. We built a framework for evaluating urban spatial identity based on the relationships between climate characteristics and urban morphological characteristics. Then, we conducted an empirical study in five small cities. Through the work in our study, we look forward to developing a macroscopic and systematic grading method for urban spatial identity.

## 2. Materials and Method

### 2.1. Study Area

**Figure 2:**
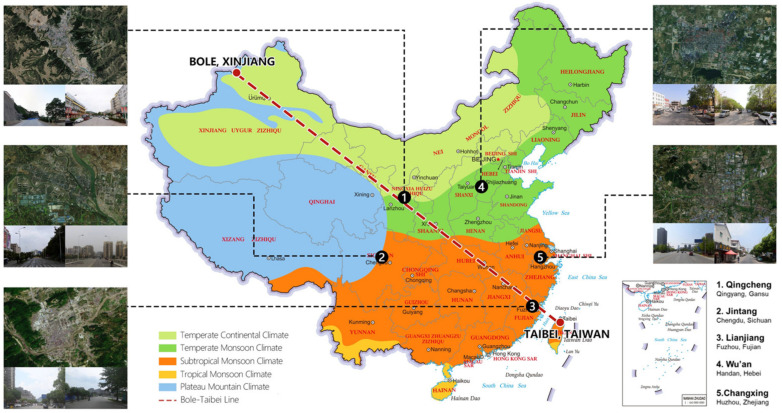
Location of observation cities.

Our study was conducted in central urban areas of small cities (with fewer than 500,000 residents within city boundaries). Our observation cities were from both sides of China’s regional development balance line and are also in two different climate zones (hot humid, and cold) based on China’s building climate zoning. Five cities were selected in two different climate zones: Wu’an, in Hebei Province; Qingcheng, in Gansu Province; Jintang, in Sichuan Province; Changxing, in Zhejiang Province; and Lianjiang, in Fujian Province (see Figure 2).

These case areas were also considered due to their geographic features. Plateaus and plains dominate the terrain in the cold climate zones of China. Qingcheng is located on the Loess Plateau, while Wu’an is in the North China Plain. Basins, plains, and hills dominate the terrain in China’s hot humid climate zones. Jintang is located in the Sichuan Basin, Changxing in the Middle and Lower Yangtze River Plain, and Lianjiang in the Southeast Hills.

In the specific selection of small cities, we considered differences in urbanization rates. In relevant studies, they significantly influenced urban spatial identity in China [8]. We selected areas with higher and lower urbanization rates in both climate zones to increase the scientific validity of the comparison between groups in the different climate zones. Specifically, the ratio of the highest and lowest urbanization rates in the two climate zones is similar.

Five central urban areas were divided into blocks based on roads and other physical boundaries. We conducted a three-step block-selection procedure to guarantee sample data’s availability and representativeness.

The first step was to explore the spatial characteristics of blocks. Forms and placement features were used to perform the spatial characteristics of blocks, including three drivers: roundness, distance from water, and relationship with the old urban area.

The second step was to explore blocks’ dominant functions, which reflect the functional characteristics of each block. There were seven categories, including productivity, life, ecology, and four different hybrid types (productivity–life, productivity–ecology, life–ecology, and productivity–life–ecology) of integrated functions.

**Figure 3:**
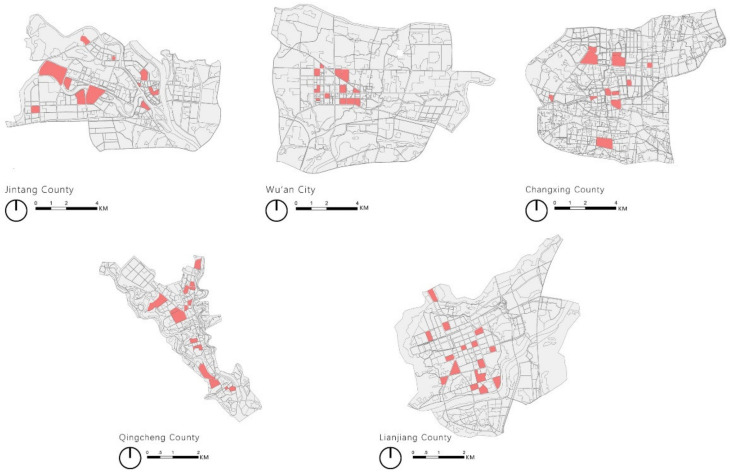
Samples from 481 blocks in five cities.

The third step was to obtain a block sample according to the proportion of functional and spatial categories. As shown in Figure 3, there are samples from 481 blocks in five cities, comprising 60 blocks (12 in each city).

### 2.2. Data Sources

The major meteorological datasets of the five cities from 2009–2017, including mean annual temperature, humidity, etc., were used. These were provided by local statistical bureaus and meteorological bureaus. Some missing data were supplemented through the China Meteorological Data Network (http://data.cma.cn/site/index.html, accessed on 25 October 2021).

The major morphological datasets for the central urban areas of the five cities in 2017 were used. This part of the data was mainly derived from topographic maps and basic planning data provided by the respective construction bureaus, natural resources, and planning bureaus. Some missing data were supplemented through the National Platform for Common Geospatial Information Services (https://map.tianditu.gov.cn/, accessed on 25 October 2021).

### 2.3. Methods

#### 2.3.1. Research Framework

Based on the theoretical research, we considered that urban spatial identity could be analyzed regarding the relationship between climate and space. Therefore, we used the coupling coordination model to measure the degree of coordination between the morphological characteristics and the regional climate. The coupling coordination model is a common approach used to analyze the coupled relationships between different systems, which include urban space, human behavior, and eco-environment [33,34]. Urban development activities are mainly associated with factors such as natural resources and cultural background, which are closely linked to the regional climate [35,36]. On the other hand, a block is a controlling unit for implementing space control in China [37]. Previous research also suggests that the spatial form at a block scale potentially influences climate conditions [21]. Therefore, the relationship between urban regional climate and the block’s spatial pattern is analyzed in our research.

**Figure 4:**
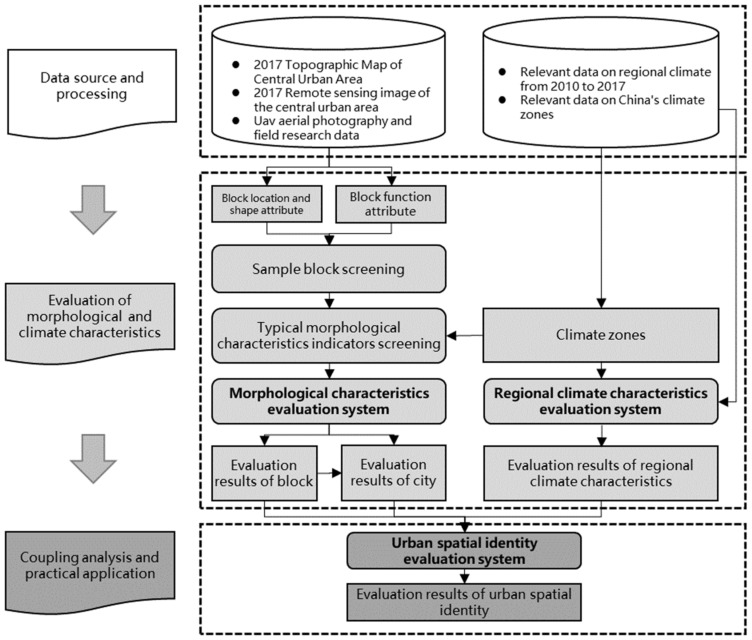
The framework for evaluating the spatial identity.

We constructed a research framework (see Figure 4) to demonstrate the entire workflow. In this framework, the evaluation of spatial identity requires the comprehensive evaluation indexes of regional climates and blocks’ morphological systems. Specifically, the framework includes three parts: the evaluation of regional climate characteristics, the evaluation of morphological characteristics, and the evaluation of spatial identity (see Figure 4). In the following subsections, we will describe the specific assessment steps and the statistical and mathematical tools.

#### 2.3.2. Evaluation of Regional Climate Characteristics

We used urban planning and human-comfort-related indicators of regional climate features to evaluate regional climate characteristics. Furthermore, the entropy weighting method was used to compute the weight of climate indicators.

Firstly, we conducted standardized processing for indicators to eliminate the dimensionality effect among indicators. The equations of the standardized method adopted in this study are as follows:

(1) $$\mathrm{Positive}\;\mathrm{indicator}:\;T_{ij}=\frac{t_{ij}-min\left(t_{i}\right)}{max\left(t_{i}\right)-min\left(t_{i}\right)}$$

(2) $$\mathrm{Negative}\;\mathrm{indicator}:\;T_{ij}=\frac{max\left(t_{i}\right)-t_{ij}}{max\left(t_{i}\right)-min\left(t_{i}\right)}$$

where $T_{ij}$ represents standardized value, and $t_{ij}$ represents original value.

The entropy weighting method determines the weight of each indicator based on dispersion degrees. The higher the dispersion of an indicator, the higher the weighting of that indicator [38]. This method contributes to overcoming the subjectivity of human weighting and information overlap between multiple indicators [39,40]. Equations are as follows:

(3) $$f_{ij}=x_{ij}^{\prime }/\overset{n}{\underset{j=1}{\sum }}x_{ij}$$

(4) $$e_{j}=-1/\ln n\sum_{j=1}^{n}f_{ij}\ln(f_{ij})\;(\mathrm{When}\;f_{ij}=0,\;e_{i}=0)$$

(5) $$g_{j}=1-e_{j}$$

(6) $$\omega_{j}=g_{i}/\;\overset{m}{\underset{i=1}{\sum }}g_{i}\;\left(0\leq \omega_{i}\leq 1\right)$$

where: $f_{ij}$ represents the proportion of the indicator; $x_{ij}^{\prime }$ represents the standardized matrix; $x_{ij}$ represents the original matrix; *n* represents the number of samples; $e_{j}$ represents the entropy of indicator; $\omega_{i}$ represents the entropy weight of indicator; $g_{i}$ represents the standard coefficient; and m represents the number of evaluation indicators.

Combining Equations (1)–(6), the weights of each climate characteristic indicators were obtained. Then, we calculated the degree of the cold and hot humid climate characteristics in the five cities from 2007 to 2017. Finally, multi-year average values for each city were used for the regional climate characteristics.

#### 2.3.3. Evaluation of Morphological Characteristics

There were three steps for evaluating urban morphological characteristics. The first step was to select urban morphological analysis dimensions and indicators that are often involved in urban planning based on relevant research. The second step was to select indicators that could reflect the differences in the spatial patterns between different climate zones. In this step, we separated five small cities into two categories based on their regional climate features. Then, we used the Mann–Whitney U test to analyze whether there were significant statistical differences in morphological indicators between the hot humid climate zones and the cold climate zones. Indicators with significant statistical differences were considered to be typical indicators that reflected morphological characteristics under the influence of climate. The last step was to conduct an evaluation method to compute the morphological index for each representative block. In this step, the process of calculation and the formulas were essentially the same for the evaluation of regional climate characteristics. First, we standardized the indicators and used the entropy weighting method to assign weights to the indicators. Then, we calculated the degree of morphological characteristics for each block in different cities. The mean value of the morphological characteristics for each block reflected the value of the morphological characteristics for each city.

#### 2.3.4. Evaluation of Urban Spatial Identity

The coupling coordination model was used to assess urban spatial identity. The couple degree, which reflects the strength of interactions between systems, is used in this study to reflect the strength of interactions between climatic and spatial morphological systems. The coupling coordination degree, which reflects the degree of coordination of intersystem interactions, is used in this study to assess the level of spatial identity. The higher the degree of coordination between climate and morphological characteristics, the higher the level of urban spatial identity. Referring to existing studies [41,42], the calculation formula is

(7) $$C={\left\{\left(U_{1}\times U_{2}\right)/{\left[\left(U_{1}+U_{2}\right)/2\right]}^{2}\right\}}^{k},\;C\in \left[0,\;1\right]\;$$

(8) $$T=\alpha \times U_{1}+\beta \times U_{2}\;$$

(9) $$D=\sqrt{C\times T},\;D\in \left[0,\;1\right]\;$$

where *C* represents the system coupling degree between climate characteristics and morphological characteristics; $U_{1}$ represents the climate characteristic evaluation index; and $U_{2}$ represents the morphological characteristic evaluation index. Previous research suggests that *k* = 2 can ensure good numerical distribution for the couple degree and the coordination degree [42]. Thus, *k* = 2 was selected in this paper for subsequent calculations. *T* represents the harmonic index. *α* and *β* are the undetermined coefficients, and *α* + *β* = 1. In this study, *α* = *β* = 0.5, due to both systems being equally important. *D* is the coupling coordination degree.

The evaluation results of the morphological characteristics and the evaluation results of the climatic characteristics were applied in this evaluation method. We used this evaluation system to derive the level of spatial identity. Moreover, we compared the levels of special identities indifferent cities and attempted to select representative blocks with a high level of spatial identity.

## 3. Results

### 3.1. Evaluation Results of Climate Characteristics

We summarized the climatic factors closely related to urban planning and human comfort. Then, we selected five dimensions of the climate factors that affect the living environment: temperature, precipitation, sunshine, wind speed, and humidity, for a total of nine quantitative indicators. We determined which of the two different evaluation objectives the positive and negative indicators were based on: hot and humid or cold climate characteristics. Table 1 shows the weights of the indicators, which were derived using the entropy weighting method.

By using China’s building climate regionalization, five cities—Changxing, Lianjiang, Jintang, Qingcheng, and Wu’an—were determined to be in two major climate zones (the cold climate zones and the hot humid climate zones).

**Figure 5:**
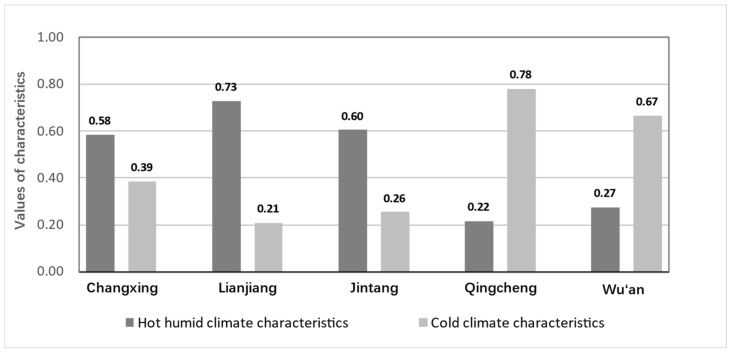
Evaluation results of the regional climate characteristics.

The composite index of climate characteristics was calculated by using 10-year average assessment values for each city (see Figure 5). In general, the values of the climate characteristics varied considerably between the cities in different climate zones. The results also showed that Lianjiang, Jintang, and Changxing, which are located in the hot humid climate zones, performed better higher regarding the hot humid climate characteristics than the other cities. Lianjiang had the highest hot humid climate characteristics index, followed by Jintang and Changxing. Similarly, Wu’an and Qingcheng, which are located in the cold climate zones, performed significantly better regarding the cold climate characteristics than the other cities. Furthermore, there was a higher cold climate characteristics index in Qingcheng than in Wu’an.

### 3.2. Evaluation Results of Morphological Characteristics

#### 3.2.1. Morphological Characteristics Evaluation System

The morphological characteristics evaluation system included the morphological indicators usually used in urban planning [43,44]. There are twelve indicators in four dimensions: block shape, land cover, spatial order of buildings, and development density (see Table 2).

The morphological data of the five cities were divided into two groups. The first group was compatible with the climate characteristics in the hot humid climate zones, named Group H. The second group was compatible with the climate characteristics in the cold climate zones, named Group C. Then, we used the Mann–Whitney U test to assess whether there were significant statistical differences in the morphological indicators of urban blocks between Group H and Group C. Table 3 shows that the data distributions of the impervious surface rate, green coverage rate, water surface rate, average story number, and total gross floor area differ between the groups (*p* < 0.05). Therefore, these morphological indicators were chosen as representative indicators to construct an evaluation system for the morphological characteristics.

**Figure 6:**
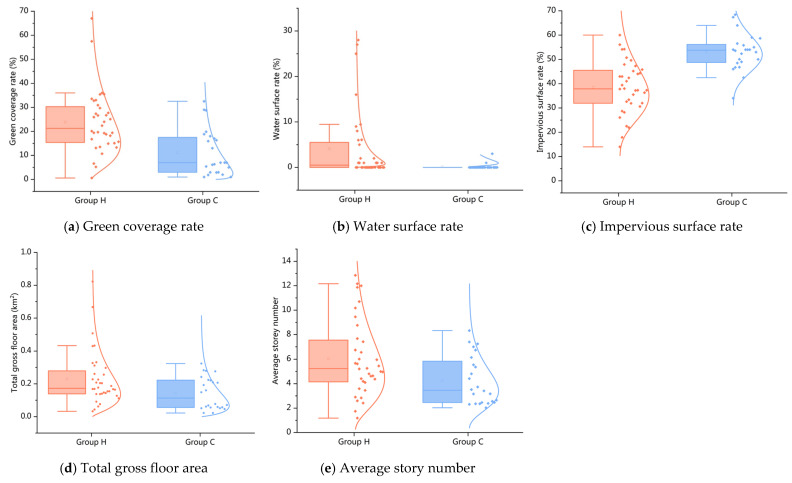
Comparison of typical morphological indicators in different climate zones.

Figure 6 shows the distribution of each typical indicator, including the green coverage rate (Figure 6a), water surface rate (Figure 6b), total gross floor area (Figure 6d), and average story number (Figure 6e), which are significantly higher in Group H. In contrast, the impervious surface rate (Figure 6c) is relatively higher in Group C. Furthermore, the data distributions for water surface rate and impervious surface rate were considerably different between groups. Group H is prone to outliers in several indicators compared to Group C.

Consequently, we determined the positivity or negativity of the indicators for evaluating the morphological characteristics in the different climate zones according to the results of the comparison between groups. Furthermore, the determination of the morphological indicators’ weighting was also based on two different evaluation objectives: the morphological characteristics that are compatible with the hot humid climate or the cold climate characteristics. Then, the indicator weights were calculated using the entropy weighting method (Table 4).

#### 3.2.2. Evaluation Results of Morphological Characteristics

We conducted an evaluation for each block. For Group H, the results showed that multiple blocks with high values for the “morphological characteristics that are compatible with the climate of hot humid climate zones” were found in both Jintang and Lianjiang. For Group C, the results showed that multiple blocks with high values for the “morphological characteristics that are compatible with the climate of cold climate zones” were found in Qingcheng. In contrast, Wu’an had some blocks with high values of morphological characteristics, and the distribution of the morphological characteristics data was more fragmented across Wu’an.

**Figure 7:**
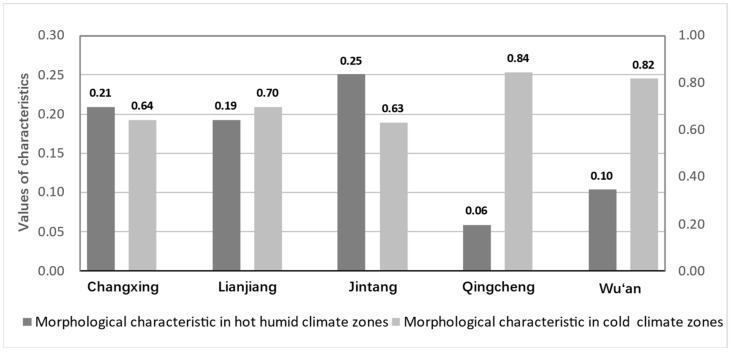
Evaluation results of morphological characteristics.

Then, the composite index of the morphological characteristics was calculated by the blocks’ average assessment values for each city (see Figure 7). Overall, the differences in the values of morphological characteristics compatible with the hot humid climate zones were more significant across cities. In contrast, there was less variation between cities for the degree of the morphological characteristics compatible with the cold climate zones. The results showed that Jintang had the most prominent morphological characteristic value in Group H, followed by Changxing. In contrast, Lianjiang had the lowest value. In Group C, Qingcheng had a higher morphological characteristic value than Wu’an.

### 3.3. Evaluation Results of Urban Spatial Identity

#### 3.3.1. Evaluation Results of Central Urban Areas’ Spatial Identity

**Figure 8:**
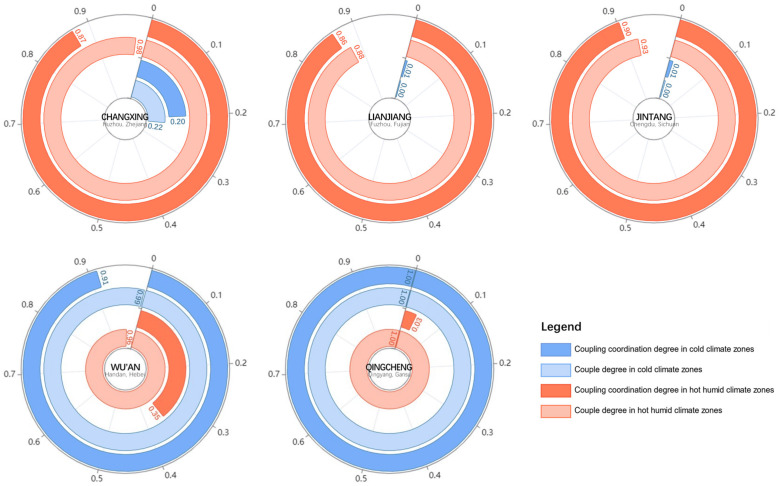
Evaluation results of spatial identity in central urban area of each city.

Combining climate characteristics evaluation and morphological characteristics evaluation, we conducted an evaluation of the spatial identity (see Figure 8). Based on the evaluation methodology constructed in our research, the coupling coordination degree was used to reflect the level of “urban spatial identity based on climate adaptation”. Jintang had the highest spatial identity value of the hot humid climate zones, followed by Lianjiang and Changxing. The results also showed a high strength of interaction between the climate and morphological systems in Changxing, followed by Jintang and Lianjiang. Qingcheng had a higher spatial identity value for the cold climate zones than Wu’an but was similar in terms of the intensity of interaction. Notably, both types of couple degrees were high in Wu’an and Qingcheng. Furthermore, Wu’an is in the cold climate zones but had a slight spatial identity value with the hot humid climate zones in our study. Moreover, Changxing is in the hot humid climate zones, which have a slight spatial identity with the cold climate zones.

#### 3.3.2. Representative Blocks Screening

**Figure 9:**
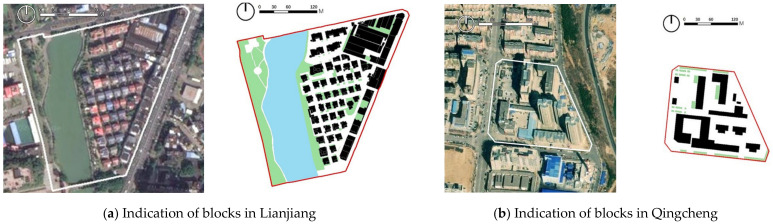
Typical blocks in different cities.

The most distinctive blocks were chosen by the coupling coordination degree. In the hot humid climate zones, some blocks in Lianjiang and Jintang had higher spatial identity values. Figure 9a shows one of the most typical blocks in Lianjiang, which is dominated by low-rise and medium-rise residential and commercial complexes and a significant proportion of water and public green space. In the cold climate zones, the spatial identity values of blocks in both Qingcheng and Wu’an were high overall. Figure 9b shows one of the most typical blocks in Qingcheng. There is a significant amount of public city space, and inside the buildings are primarily high-rise residential, hotels, and medical facilities. In general, this suggests a reference for establishing urban spatial identity by finding the blocks with the highest levels of spatial identity in each city.

## 4. Discussion

### 4.1. Typical Indicators of Urban Morphological Characteristics

We found five morphological indicators that differ considerably between hot humid climate zones and cold climate zones. They coincide with some geometric and surface-cover indicators typical of “local climate zones” (LCZ) [45]. They are mainly subordinate to two dimensions: land cover and urban development density.

For the dimension of land cover, green coverage rate and water surface rate had significant characteristics in the hot humid climate zones. An apparent and direct reason for this is the relative abundance of precipitation in the hot humid climate zones. A suitable temperature, humidity, and sunlight are all conducive to plant growth. Cold climate zones with relatively poorer climatic conditions require more maintenance costs for greenery and water and limit their share. Another possible reason is that the public also tends to use water features and plant trees to improve the outdoor thermal environment in hot humid climate zones [46]. On the other hand, the impervious surface rate was higher in cold climate zones compared to hot humid climate zones. To ensure a more comfortable temperature in winter, residents in cold climate zones have a high demand for more direct sunlight [47,48], which may lead to more open space with less tree cover and contribute to the high impervious surface rates.

For the dimension of urban development density, the total gross floor area and average story number were high in hot humid climate zones. Government policies related to climate conditions may also have an impact. For example, the daylighting standard in cold regions is more stringent, leading to construction scale control. Further, for a long time, central heating charges in cold areas of China have been based on the floor area of the house. The cost of heating may also have placed a limit on the floor area.

However, the cold climate zones of some cities had ambitious ecological construction goals. Related research also points to an over-greening of northwestern and northeastern China [49]. Although we need to resist over-greening, the effect of urban greening on improving people’s physical and mental health cannot be ignored [50,51]. Therefore, the cold climate zones of cities might show potential for increasing water and greenery rates in the future if the effects of the two trends deepen further. For the first trend, China’s cold climate zones are significantly affected by global warming and increased summer temperatures [52], contributing to higher outdoor temperature regulation needs for residents in colder areas during the summer. For the second trend, warming through urban heat islands in cold climate zones significantly lengthens the growing season [53], suggesting that a wider variety and number of plants can persist.

### 4.2. Evaluation of Urban Identity

Our findings suggested that Jintang, followed by Lianjiang and Changxing, had the highest degree of spatial identity in the hot humid climate zones. Furthermore, Qingcheng had a higher spatial identity than Wu’an in the cold climate zones.

Related research demonstrates that China’s urbanization process negatively impacts urban spatial identity [8]. There is a phenomenon in China’s urbanization that “delay in building culture and environment in the city compared to the actual construction” [54]. This situation is reflected in many aspects of urban construction in China, such as the destruction of natural and cultural resources, destructive construction, and repetitive urban and architectural forms [8]. Our findings suggest similar results: (1) In the hot humid climate zones, Changxing’s urbanization rate was 58.5% in 2017, which was significantly higher than Lianjiang’s (46.1%) and Jintang’s (42.1%). Of these, Jintang had the lowest urbanization rate but the highest level of spatial identity. (2) In the cold climate zones, the urbanization rate in Wu’an was 50.55% in 2017, which is higher than Qingcheng’s (36.5%). Qingcheng has the lowest urbanization rate but the highest level of spatial identity.

In addition, it is noteworthy that Lianjiang had a comparable level of spatial identity to Changxing but a significantly lower urbanization rate, suggesting that its spatial identity will be predictably under threat in the future. Our research also selected blocks with a high degree of spatial identity. We may conduct proper planning, including introducing natural elements and managing the intensity of construction development, to enhance the spatial identity of the block scale to some degree.

Furthermore, Changxing and Wu’an had different spatial identities based on their local climate characteristics, which may be the reason for including two parts. For the first part, there was a negative consequence of a higher degree of urbanization, leading to a decline in spatial identity. For the second part, there was an influence by the local seasonal climatic characteristics. We found that the climatic conditions in summer in Wu’an were like those of some cities in the hot humid climate zones, such as temperature, humidity, and other indicators. Moreover, a similar situation existed in Changxing during the winter. These results suggested that we should consider seasonal changes for residents’ demand for spatial form regarding demands for urban spatial identity under climate adaptation.

### 4.3. Limitations

Some limitations may be present in our study. Firstly, the affecting systems should be investigated more thoroughly to enhance the precision and universality of the theoretical model and evaluation findings. A city can be considered a complex system, and multiple systems influence the urban form [55]. However, we only explored urban spatial identity from a climatic perspective. The more that is revealed about the influencing factors and mechanisms of the actions of spatial morphological systems, the broader the perspective of the research on spatial identity could be. For instance, synchronizing the natural environment and socioeconomic systems might make the evaluation system more accurate and comprehensive. Secondly, the selection of climatic and morphological dimensions and indicators was insufficient. In terms of the climate characteristic indicators, there was both a neglect of climate extremes such as drought and heavy rainfall and a lack of attention to seasonal variations. These may influence human behaviors and construction activities and, thus, alter spatial patterns [56]. The morphological indicators were also lacking in describing the morphological details at the block scale. Finally, the bidirectional regulatory link between climate and spatial systems is a long-term, dynamic process. However, we did not expand on the details of the long-term effects of the two systems. Exploring the synergistic changes in the spatial patterns of urban blocks and regional climate over time may enable researchers to provide a more scientific understanding of urban characteristics in the context of climate adaptation. However, the analysis of the dynamic coupling between spatial morphology and climate in small cities in this study was deficient due to the difficulty of obtaining data on the morphological characteristics of small cities over time. In addition, the case areas selected for our research were mainly in the cold and hot humid climate zones, but there are more diverse types of climate zones to be studied both within China and globally. The applicability of our evaluation framework to cities in multiple climate zones also needs to be improved.

## 5. Conclusions

We developed a framework for assessing the spatial identity of central urban areas and used this framework to find the urban spatial identity of five cities in two different climate zones in China. The results can provide the basis for comparing the spatial identities of cities in hot humid and cold climate zones, identifying areas where the spatial identities need improvement, and providing a reference to shape urban spatial identities during the stages of urban development and regeneration.

Furthermore, to address the limitations of our research, we will consider choosing an optimal exact index system in future studies. We will explore the dynamic coupling of the three subsystems of regional context, human activities, and spatial patterns and construct an evaluation model of spatial identity accordingly, to determine the spatial identity more scientifically and precisely.

## Figures and Tables

**Table 1:** Weighting of indicators for the evaluation of regional climate characteristics.

Dimension	Indicator	Indicator Properties of Hot humid Climate Characteristics	Indicator Weight of Hot humid Climate Characteristics	Indicator Properties of Cold Climate Characteristics	Indicator Weight of Cold Climate Characteristics
Temperature	Annual mean air temperature	Positive	0.116	Negative	0.188
	Annual mean maximum temperature	Positive	0.084	Negative	0.041
	Annual mean minimum temperature	Positive	0.119	Negative	0.243
Rain	Annual precipitation	Positive	0.243	Negative	0.129
	Annual number of rainy days	Positive	0.094	Negative	0.059
Sunshine	Annual mean sunshine duration	Negative	0.098	Positive	0.085
Wind speed	Annual mean wind speed	Negative	0.101	Positive	0.135
Humidity	Annual mean relative humidity	Positive	0.144	Negative	0.117

**Table 2:** Common dimensions and indicators for morphological evaluation.

Dimension	Indicator	Indicator Description	Quantification Method
Block shape	Total land area (TLA)	Total land area of a sample block.	/
	Compactness ratio (CR)	Compactness is a measure of the shape characteristics of a block.	$CR=\sqrt{\pi S_{t}}/P$ where $S_{t}$ is the total land area, *P* represents the block perimeter.
Land cover	Building density (BD)	A rate of the building area to the total land area within the block.	$BD=S_{b}\;/S_{t}$ where $S_{b}$ is the building area, $S_{t}$ represents the total land area of the block.
	Green coverage rate (GCR)	A rate of green coverage area to the total land area within the block.	$GCR=S_{g}\;/S_{t}$ where $S_{g}$ is the is the green coverage area, $S_{t}$ represents the total land area of the block.
	Water surface rate (WSR)	A rate of the water surface area to the total land area within the block.	$WSR=S_{w}\;/S_{t}$ where $S_{w}$ is the water surface area, $S_{t}$ represents the total land area of the block.
	Impervious surface rate (ISR)	A rate of the impervious surface area to the total land area within the block.	$ISR=S_{i}\;/S_{t}$ where $S_{i}$ is the impervious surface area, $S_{t}$ represents the total land area of the block.
Spatial order of buildings	Degree of building angle disorder (DAD)	The mean value of the angle difference of building orientation. It is used to indicate the characteristics of the building’s directionality.	$DAD=AVERAGE\overset{n}{\underset{i=1}{\sum }}\left|A_{i}\right.-\left.A_{a}\right|$ where $A_{i}$ is the orientation angle of the building, $A_{a}$ represents the angle difference from the adjacent buildings.
	Degree of building area disorder (DBA)	The mean difference of the building area. It is used to indicate the variation degree of the building area in the block.	$BAD=AVERAGE\overset{n}{\underset{i=1}{\sum }}\left|S_{ib}\right.-\left.S_{ab}\right|$ where $S_{ib}$ is the area of the building, $S_{ab}$ represents the area of adjacent buildings.
	Degree of building distance disorder (DBD)	The standard deviation of the minimum distance. It is used to indicate the degree of variation in the spacing of buildings within a block.	$DBD=\sqrt{1/n\overset{n}{\underset{i=1}{\sum }}{\left(D_{i}-\mu \right)}^{2}}$ where $D_{i}$ is straight-line distance between the geometric centers of adjacent buildings, μ represents the arithmetic means of the distance.
Development density	Total gross floor area (TGFA)	A sum of the horizontal areas of each floor of a building.	$\mathrm{TGFA}=N_{s}*S_{b}$ where $N_{s}$ is the average story number, $S_{b}$ represents the building area.
	Average story number (ASR)	Average number of stories of all buildings in the block.	/
	Floor area ratio (FAR)	A ratio of the total gross floor area to the total area within the block.	$FAR=S_{f}\;/S_{t}$ where $S_{f}$ is the total gross floor area, $S_{t}$ represents the total land area of the block.

**Table 3:** Results of Mann–Whitney U test of morphological indicators.

Indicators of Difference Statistically Significant	Statistically Significant	Indicators of Difference Not Statistically Significant	Statistically Significant
Impervious surface rate	0.000 **	Total land area	0.194
Green coverage rate	0.000 **	Building density	0.546
Water surface rate	0.001 **	Compactness index	0.424
Average story number	0.019 **	Degree of building angle disorder	0.330
Total gross floor area	0.040 **	Degree of building distance disorder	0.556
		Degree of building distance disorder	0.056 *
		Floor area ratio	0.097

* *p* < 0.1, ** *p* < 0.05.

**Table 4:** Weighting of indicators for evaluating the morphological characteristics.

Dimension	Indicator	Indicator Properties of Group H	Indicator Weight of Group H	Indicator Properties of Group C	Indicator Weight of Group C
Development density	Total gross floor area	Positive	0.127	Negative	0.145
	Average story number	Positive	0.143	Negative	0.145
Land cover	Green coverage rate	Positive	0.108	Negative	0.187
	Water surface rate	Positive	0.573	Negative	0.195
	Impervious surface rate	Negative	0.049	Positive	0.327

## Data Availability

Not applicable.
